# Association between Number of Teeth and Chronic Systemic Diseases: A Cohort Study Followed for 13 Years

**DOI:** 10.1371/journal.pone.0123879

**Published:** 2015-05-06

**Authors:** Kolade Oluwagbemigun, Thomas Dietrich, Nicole Pischon, Manuela Bergmann, Heiner Boeing

**Affiliations:** 1 Department of Epidemiology, German Institute of Human Nutrition, Potsdam–Nuthetal, Germany; 2 Department of Oral Surgery, the School of Dentistry, University of Birmingham, Birmingham, United Kingdom; 3 Department of Periodontology, Charité–Universitätsmedizin Berlin, Berlin, Germany; National University of Singapore, SINGAPORE

## Abstract

**Background:**

There is growing evidence of an association between oral health, specifically dental status, and chronic systemic diseases. However, varying measures of dental status across different populations and low study sample has made comparison of studies and conclusion of findings unclear. Our aim is to examine whether the number of teeth as a measure of dental status is associated with incident chronic diseases in a cohort setting.

**Methods:**

We conducted a cohort study among 24,313 middle-aged Germans followed up for 13 years. Data on number of teeth as a measure of dental status were obtained through self-reports. Outcomes were clinically–verified incident non–fatal myocardial infarction, stroke, type 2 diabetes mellitus, and cancer. Hazard ratio (HR) and 95% confidence intervals (CI) were obtained from Cox regression models.

**Results:**

Increasing number of teeth is inversely related to risk of myocardial infarction (HR: 0.97; 95% CI: 0.96, 0.99). The full multivariate model of teeth groups showed a strong linear trend for myocardial infarction, a less strong trend for stroke, and no relation with type 2 diabetes mellitus and cancer in a competing risk model. Participants with 18–23 teeth and those without teeth were at 76% (95%CI: 1.04, 3) and 2.93 times (95%CI: 1.61, 5.18) higher risk of myocardial infarction compared to those with nearly all teeth (28–32 teeth).

**Conclusions:**

Number of teeth is specifically associated with myocardial infarction and not with other chronic disease indicating that dental status further strengthens the link between oral health and cardiovascular diseases.

## Introduction

Epidemiologists consistently opined association between measures of dental status and chronic systemic diseases, especially those with prominent inflammatory components such as cardiovascular disease (CVD), type 2 diabetes mellitus (T2DM) and cancer [1]. Such measures include history of periodontitis, periodontal pocket depth, clinical attachment loss, tooth loss, number of teeth and dental indices. There is growing evidence linking some of these measures of dental status to myocardial infarction (MI), stroke, T2DM and cancer, although most of this evidence is from case–control and cross–sectional studies [2–7]. Cohort studies have linked dental status to specifically atherosclerotic CVD [8–10], cerebral and ischaemic stroke [11, 12] and oropharyngeal cancers [2, 13]. Some explanations for these associations include low–grade systemic inflammation that accompanies local periodontal inflammation, tooth loss resulting in dietary change and inadequate adjustments for confounding by smoking and socioeconomic status (SES) [14].

However, evidence from European cohort studies [14–20] which are relatively smaller when compared to those among American and Asian cohorts are less convincing.Therefore the relationship between dental status and risk of MI, stroke, T2DM and cancer among European populations is not yet fully resolved and as such requires more evidence. Furthermore, studies among Europeans have focused on single chronic systemic disease outcome or closely–related diseases. It is therefore important to investigate these findings in larger–scale prospective studies across multiple chronic systemic disease end points which can be competing outcomes. Such studies will ensure previous evidence is put in better perspective thereby raising potential research questions regarding pathogenesis. A vital consideration in such studies should be that the measure of dental status is reliable and easily accessible. One of such measure is self–reported number of teeth [21, 22].

The aims of this present cohort study are to investigate whether participants with lower number of teeth have a higher risk of MI, stroke, T2DM and cancer; whether the relationship is specific for only one of these diseases and if so, whether such relationship is independent of smoking, SES, other established risk factors and diet.

## Materials and Methods

### Study population

The study population is established within the Europe–wide Prospective Investigation into Cancer and Nutrition–Potsdam (EPIC–Potsdam). EPIC–Potsdam is a cohort study that is part of the multicentre EPIC study. EPIC–Potsdam comprises 27 548 middle-aged (35–64 years old) participants recruited between 1994 and 1998 from the general population of Potsdam, Germany and adjacent communities from the general population registries. Boeing et al [23] presents details of study design and recruitment. Ethical approval was obtained from the ethics committee of medical society of the federal state of Brandenburg, Germany and all participants gave written informed consent for participation in the study.

At baseline, body mass index (BMI) was calculated from body weight and height which were measured at the study centre. Other information were collected through questionnaires and interview: age, sex, alcohol consumption (quantity per day) and smoking status (non–smokers, current smokers [numbers of cigarettes smoked per day, smoking duration] and time since cessation for former smokers). Educational attainment was expressed as vocational school or less, technical school and university. Vocational school or less implies a lower education of 10 years of school education with 2 years of additional professional training. Technical school implies 10 years of school followed by more than 2 years of professional training. Occupation was defined as higher–grade professionals, lower–grade professionals, skilled manual worker or non–manual employed and simple manual workers. Physical activity information was obtained through a plethora of questions which were used to assign participants to one of four categories of the Cambridge physical activity index (inactive, moderately inactive, moderately active and active). Dietary factors were assessed by a validated self–administered 148–item food frequency questionnaire (FFQ), aggregated into 49 separate food groups. Intake of antibiotics, vitamin and/or mineral supplements, non–steroidal anti–inflammatory drugs (NSAID) and hormone replacement therapy (HRT) `were assessed by self–reports from interview and FFQ. Prevalent MI, stroke, T2DM, cancer, angina pectoris, heart failure and transient ischaemic shock were self–reported and validated by a study physician using medical record review. Prevalent hypertension was defined as systolic blood pressure greater than 140 mm Hg or diastolic blood pressure greater than 90 mm Hg or self–reporting of a diagnosis or use of antihypertensive medication. The cohort was followed by follow–up waves in 2–year intervals. At fourth follow–up (between 2004 and 2006), participants provided information from questionnaire on number of teeth (“How many natural teeth do you have?”) and number of teeth lost since baseline (“How many teeth have you lost since your visit to the study centre?” and the date of the visit was given). Information on history of periodontitis and bone loss in the mouth was also provided (“Has a dentist ever told you that you have periodontitis and /or that you are losing (or have lost) the bone around your teeth?”).

Information on incident chronic diseases was collected using follow-up questionnaires, data linkages with the local hospital including subsequent medical verification with the participant's physician [24].

### Data analysis

We calculated the number of teeth at baseline by adding the number of teeth lost during the follow–up period to the number of teeth present at the fourth follow–up. After excluding those with missing data on teeth status (n = 3 234) and those with more than 32 teeth (n = 1); the final analytic sample comprised 24 313 participants (14 953 women and 9 360 men). To compare participants, an a posteriori group of number of teeth (0, 1–17, 18–23, 24–27, and 28–32) was made with a reference group that has largest sample size and a separate group for full edentates (no teeth). These groups were characterized by descriptive statistics in order to examine univariate correlates of number of teeth. Basic characteristics of the study population were expressed as the arithmetic mean (standard deviation) or median (interquartile range) for continuous variables and number, percentages for categorical variables. Kruskal Wallis and Chi-square tests were used to confirm statistically significant differences in continuous and categorical variables respectively.

To determine whether number of teeth is associated with MI, stroke, T2DM and cancer; firstly we checked if there is interaction between number of teeth and age group (< 50years and ≥50years), sex, BMI group (<18–25 kg/m2, >25–30 kg/m2, and >30 kg/m2), smoking status (non-smokers, current smokers and former smokers), history of periodontitis and bone loss in the mouth (yes or no) and prevalent T2DM (yes or no) in order to present sub–group analyses if these effects are significant.

Secondly, using Cox risk models we determined the association between number of teeth groups (0, 1–17, 18–23, 24–27, 28–32 [reference]) and MI, stroke, T2DM and cancer adjusted for covariates. Model 1 was adjusted for age (continuous), sex and BMI (continuous). Model 2 was additionally adjusted for educational attainment (vocational school or less, technical school, and university), occupation (higher–grade professionals ‚ lower–grade professionals, skilled manual worker or non–manual employed‚ simple manual workers), alcohol consumption (non–consumers; women:0–6, 6–12 or >12 g/day; men:0–12,12–24, or 24 g/day), smoking status (non–smokers, current smokers [15, 15–24, or >25 cigarettes/day, smoking duration:10, 11–20, 21–30, 31–40, or>40 years], former–smokers [gave up smoking: 10, 11–20, or >20 years]), physical activity (inactive, moderately inactive, moderately active and active), and intake of vitamin and/0r mineral supplements, antibiotics, NSAID and HRT. Model 3 was additionally adjusted for prevalent hypertension, MI, stroke, T2DM, cancer, angina pectoris, heart failure and transient ischaemic shock. For each incident chronic disease model, we excluded all prevalent cases of the disease. Model 4 was additionally adjusted for dietary risk factors by including them as three factors retained from factor analysis of all 49 food groups. In model 5, we conducted competing risk analyses for Cox regression based on model 4. We test whether there is a linear trend in each model by employing orthogonal polynomial contrast. Additionally, we conducted analyses between number of teeth on a continuous scale and each chronic disease adjusted for covariates in model 4.

Finally, using restricted cubic spline (RCS) regression we investigated non–linear associations between number of teeth and each chronic disease adjusted for all covariates in model 4 above. To avoid over–fitting, we limit RCS model to five knots at teeth number: 0, 19, 25, 28 and 32 (5, 27.5, 50, 72.5 and 95 percentiles).

For all models, we corrected for time–varying covariates by specifying linear interaction with time and validity of the proportional hazards assumption (PHA) was tested with Grambsch and Therneau method based on Schoenfeld residuals. Efron’s method was used to adjust for ties in survival time and robust standard errors were computed.

### Sensitivity analysis

We excluded edentulous participants and all models were re–analysed. We checked whether diet mediate the associations between number of teeth and each chronic disease in steps described by Frazier et al [25]. Statistical analyses were performed in SAS enterprise guide 6.1. Statistical significance was determined by a two–sided P≺0.05.

## Results

### Basic characteristics of the study population

The mean age of the study population was 50 years with women relatively younger (49 years) than men (52 years)-. Median number of teeth was 25 (25 in women compared to 26 in men). Table 1 shows that participants with fewer teeth generally were older, more likely to be current smokers but consume less alcohol, had lower physical activity, had lower educational attainment and more likely to do simple manual work. Participants with fewer teeth were also likely to have more prevalent hypertension, T2DM, MI, stroke, cancer and periodontitis. These participants also eat less quantity of whole–grain bread. In addition, post–menopausal women with fewer teeth were less likely to take HRT.

**Table 1:** Basic characteristics of the study population according to the number of teeth.

	Number of Teeth					P–value
	28–32	24–27	18–23	1–17	0	
Number of Participants	8536	5976	3738	4366	1697	
Age (years), mean (s.d)	47 (8.12)	49 (8.38)	52 (8.36)	56 (7.74)	55 (8.51)	<0.01
Women, No. (%)	5028 (58.90)	3842 (64.29)	2427 (64.92)	2676 (61.30)	983 (57.93)	NS
Post–menopause^¶^, No. (%)	715 (14.22)	776 (20.20)	809 (33.33)	1228 (45.89)	447 (45.47)	NS
Anthropometry						
BMI (kg/m^2^), mean (s.d)	25.59 (4.01)	25.84 (4.08)	26.64 (4.38)	27.19 (4.38)	27.32 (4.39)	<0.01
Education, occupation and Lifestyle factors						
Vocational school or less, No. (%)	2647 (31.01)	1996 (33.40)	1477 (39.51)	2061 (47.21)	882 (51.97)	<0.01
Simple manual worker, No. (%)	183 (2.15)	119 (1.99)	132 (3.54)	213 (4.89)	119 (7.02)	<0.01
Physical activity (active), No. (%)	1486 (17.41)	962 (16.10)	565 (15.12)	663 (15.19)	239 (14.08)	<0.01
Alcohol intake, No. (%)	8358 (97.91)	5849 (97.87)	3629 (97.08)	4196 (96.11)	1615 (95.17)	<0.01
Alcohol intake (g/dy), mean (s.d)	15.14 (19.84)	13.04 (16.72)	12.9 (17.11)	14.13 (19.59)	13.42 (18.72)	0.02
Current smokers, No. (%)	1494 (17.50)	1069 (17.89)	740 (19.80)	1009 (23.11)	424 (24.99)	<0.01
Number of cigarettes/day, ^¶^mean (s.d)	11 (10.22)	12 (9.12)	13 (9.04)	13 (9.12)	14 (9.23)	<0.01
Prevalent disease at baseline						
Hypertension, No. (%)	3516 (41.19)	2663 (44.56)	1943 (51.98)	2427 (55.59)	982 (57.87)	0.03
Type 2 diabetes mellitus, No. (%)	249 (2.92)	199 (3.33)	197 (5.27)	352 (8.06)	176 (10.37)	<0.01
Myocardial infarction, No. (%)	75 (0.88)	73 (1.22)	62 (1.66)	1.26 (2.89)	76 (4.48)	<0.01
Stroke, No. (%)	60 (0.70)	49 (0.82)	44 (1.18)	67 (1.53	29 (1.71)	0.01
Cancer, No. (%)	263 (3.08)	240 (4.02)	183 (4.90)	223 (5.11)	86 (5.07)	<0.01
Periodontitis^††^, No. (%)	1034 (12.11)	862 (14.42)	680 (18.19)	1048 (24.00)	363 (21.39)	0.03
Medication use						
Multivitamin supplements, No. (%)	624 (7.31)	447 (7.48)	332 (8.61)	284 (6.50)	112 (6.60)	NS
NSAID, No. (%)	154 (1.80)	126 (2.11)	111 (2.97)	169 (3.87)	69 (4.07)	NS
Antibiotics, No. (%)	46 (0.54)	34 (0.57)	15 (0.40)	21 (0.48)	8 (0.47)	NS
Hormone replacement therapy^#^, ^¶^No. (%)	208 (29.09)	217 (27.96)	178 (22.00)	251 (20.44)	68 (15.21)	<0.01
Diet						
Meat (g/dy), mean (s.d)	41.81 (31.42)	41.36 (28.51)	40.94 (28.76)	42.72 (29.45)	42.03 (29.1o)	NS
Whole grain bread (g/dy)*,^¶^median (IQR)	27.72 (62.67)	27.21 (59.53)	24.58(57.56)	20. 25 (53.84)	19.5 (58.32)	<0.01

¶ Percentage of women;

^††^ with bone loss in mouth;

^#^ percentage of Post-menopausal women; BMI: Body mass index, NSAID: non–steroidal anti–inflammatory drugs;

^*^: median intake, No. (%): number (percentage); mean (s.d): mean (standard deviation); median (IQR): median (interquartile range);

g/dy: grams per day; NS: non–significant (P–value > 0.05)

During a median of 8.3 years (range 1–13.1) follow–up, there were 233 (60 women and 173 men) incident cases of MI, 225 (102 women and 123 men) incident cases of stroke, 987 (422 women and 565 men) incident cases of T2DM and 1 015 (536 women and 479 men) incident cases of cancer.

### Myocardial infarction and Stroke

For MI and stroke, there was no significant interaction between number of teeth and age group, sex, BMI group, smoking status, history of periodontitis and bone loss in the mouth and prevalent T2DM. When adjusted for age, sex and BMI, participants with less than 28 teeth were at higher risk of MI compared to those with 28 teeth or more (hazard ratio (HR) 1.65, 95% CI 1.06, 2.59 for 24–27; HR 1.87, 95% CI 1.15, 3.06 for 18–23; HR 1.06, 95% CI 0.48, 2.31 for 1–17 and HR 4.2, 95% CI 2.51, 7.02 for full edentates) (Table 2, MI, model 1).

**Table 2:** Multivariate hazard ratio and 95% confidence interval of association between number of teeth, myocardial infarction and stroke.

Number of teeth				Myocardial Infarction						
HR per number of tooth			Groups	Incident cases		Model 1	Model 2	Model 3	Model 4	Model 5^†^
	0.97 (0.96–0.99)^§^		28–32	40		HR: Ref. (1.00)	HR: Ref. (1.00)	HR: Ref. (1.00)	HR: Ref. (1.00)	HR: Ref. (1.00)
P–value^*^	<0.01		24–27	44		1.65 (1.06–2.59)	1.59 (1.02–2.48)	1.59 (1.02–2.48)	1.59 (1.02–2.48)	1.61 (0.98–2.65)
			18–23	39		1.87 (1.15–3.06)	1.68 (1.03–2.73)	1.65 (1.03–2.64)	1.64 (1.02–2.64)	1.76 (1.04–3)
			1–17^§^	61		1.06 (0.48–2.31)	0.82 (0.37–1.8)	0.8 (0.37–1.74)	0.77 (0.36–1.68)	0.9 (0.4–2.21)
			0	49		4.2 (2.51–7.02)	3.12 (1.84–5.29)	2.97(1.78–4.94)	2.91 (1.74–4.86)	2.93 (1.61–5.18)
			P–value^‡^		<0.01		<0.01	0.03	0.04	0.04
Number of teeth				Stroke						
HR per number of tooth			Groups	Incident cases		Model 1	Model 2	Model 3	Model 4	Model 5^†^
	0.99 (0.97–1.01)^§^		28–32	42		HR: Ref. (1.00)	HR: Ref. (1.00)	HR: Ref. (1.00)	HR: Ref. (1.00)	HR: Ref. (1.00)
P–value^*^	0.35		24–27	34		1 (0.63–1.6)	1 (0.63–1.59)	1 (0.63–1.6)	1.01 (0.64–1.62)	0.94 (0.48–1.5)
			18–23	49		1.7 (1.09–2.66)	1.66 (1.06–2.58)	1.64 (1.05–2.55)	1.64 (1.05–2.63)	1.5(0.96–2.43)
			1–17	57		1.17 (0.54–2.56)	1.07 (0.47–2.41)	1.06(0.48–2.34)	1.06 (0.48–2.36)	0.98 (0.42–2.26)
			0	43		2.34 (1.38–3.97)	2.07 (1.21–3.54)	1.99 (1.16–3.43)	1.95 (1.13–3.39)	1.82 (0.52–3.21)
			P–value^‡^		<0.01		<0.01	<0.01	<0.01	0.09

^§^: adjusted for Age (continuous), Sex, BMI (continuous), education (3 categories), occupation (4 categories), Lifestyle (smoking (never, former, current, number of cigarettes per day), alcohol consumption (continuous), physical activity (Cambridge physical activity index), use of vitamin and/or mineral supplements, antibiotics and non–steroidal anti–inflammatory drugs, hormone replacement therapy (women), prevalent diseases and three retained factors from factor analysis of 49 food groups

Model 1: Age (continuous), Sex, BMI (continuous), 1–17 teeth×time interaction.

Model 2: Model 1+ education (3 categories), occupation (4 categories), Lifestyle (smoking (never, former, current, number of cigarettes per day), alcohol consumption (continuous), physical activity (Cambridge physical activity index), use of vitamin and/or mineral supplements, antibiotics, hormone replacement therapy (women) and non–steroidal anti–inflammatory drugs

Model 3: Model 2+ prevalent diseases (MI: prevalent hypertension, angina pectoris, heart failure, transient ischaemic shock, stroke, T2DM and cancer; stroke: prevalent hypertension, angina pectoris, heart failure, MI, transient ischaemic shock, T2DM and cancer).

Model 4: Model 3+ three retained factors from factor analysis of 49 food groups

Model 5: Model 4 + competing risk events of other three incident diseases.

HR: Hazard ratio

^*^ P–value for association;

^‡^ P–value for linear trend;

^†^ adjusted competing risk events

MI: Group 1–17teeth×time interaction (P = 0.04 for interaction) (model 4). Final model showed no evidence that proportional hazard assumption was violated (P = 0.49).

^§^Hazard ratios (unadjusted for teeth 1–17 interaction with time): 2.02 (1.26–3.23), 1.59 (1.01–2.5), 1.62 (1.01–2.59), 1.55 (0.96–2.49), 1.42 (0.9–2.52)

Stroke: All variables and final model (model 4) showed no evidence that proportional hazard assumption was violated (P = 0.11).

The HR was attenuated when education, occupation, alcohol consumption, smoking, physical activity, use of antibiotics, vitamin and /or mineral supplements, NSAIDs and HRT were added; HR changed from 1.65 to 1.59, 1.87 to 1.68, 1.06 to 0.82 and 4.2 to 3.12 respectively. When prevalent diseases and diet were added (Table 2 MI, models 3 and 4), HRs changed only slightly. The competing risk model (Table 2, MI, model 5) showed attenuation of all HR in model 4 (1.59 to 1.61, 1.64 to 1.76, 0.71 to 0.9 and 2.91 to 2.93 respectively) with only participants with 18–23 teeth and full edentates at significant higher risk of MI compared to those with 28–32 teeth. All models showed significant linear trend. Number of teeth on a continuous scale showed that for every one extra tooth there is 3% decreased risk of MI (HR 0.97, 95% CI 0.96, 0.99; P = <0.01) (Table 2, MI).

When adjusted for age, sex and BMI, only participants with 23 teeth or less were at higher risk of stroke when compared to those with 28 teeth or more (HR 1.7, 95% CI 1.09, 2.66 for 18–23 teeth; HR 1.17, 95% CI 0.54, 2.56 for 1–17 and HR 2.34, 95% CI 1.38, 3.97 for full edentates) (Table 2, stroke, model 1). The HR showed a large attenuation in full edentates (2.34 to 2.07) when education, occupation, alcohol intake, smoking, physical activity, use of antibiotics, vitamin and /or mineral supplements, NSAIDs and HRT were added (Table 2, stroke, model 2) and additional attenuation when prevalent diseases and diet were added (Table 2, stroke, models 3 and 4). In the competing risk model, no significant estimates were observed (Table 2, stroke, model 5). All models except model 5 showed significant linear trend. Number of teeth on a continuous scale showed that for every one extra tooth there is 1% decreased risk of stroke (HR 0.99, 95% CI 0.97, 1.01; P = 0.35), respectively (Table 2, stroke).

**Fig 1:**
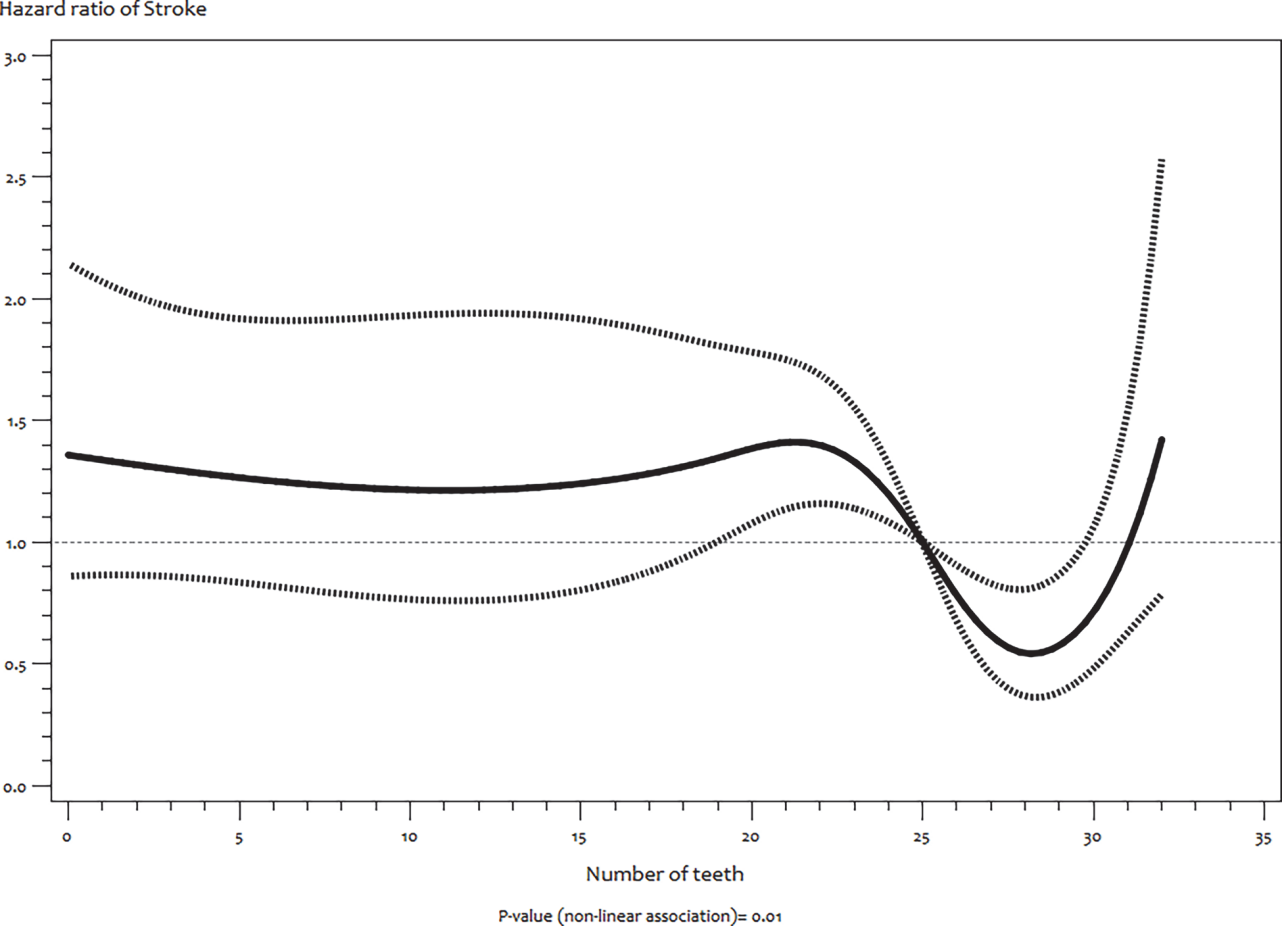
Functional relationship (and 95% pointwise confidence band) between number of teeth and hazard ratios (multivariate adjusted) of incident stroke estimated by restricted cubic splines.

**Fig 2:**
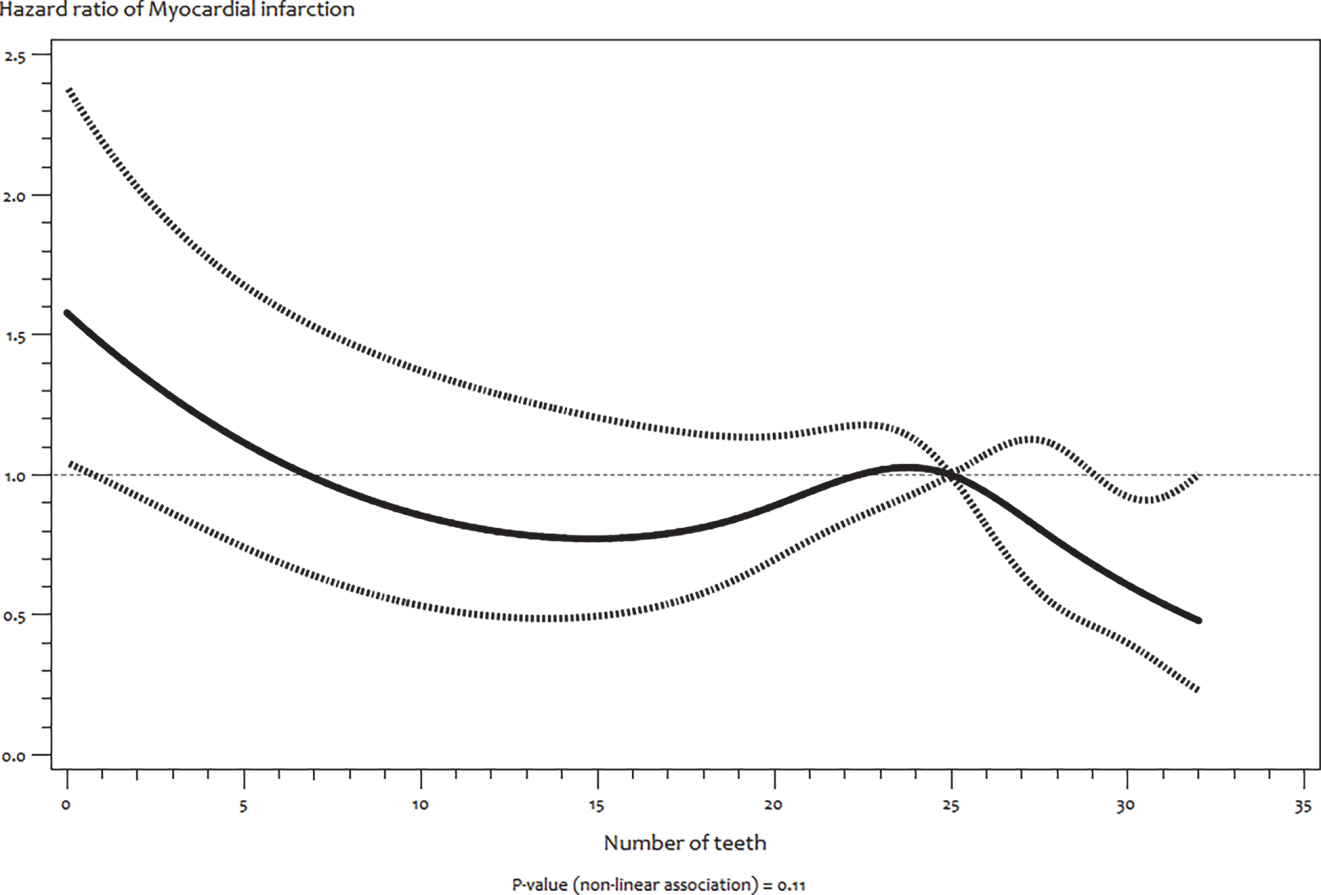
Functional relationship (and 95% pointwise confidence band) between number of teeth and hazard ratios (multivariate adjusted) of incident myocardial infarction estimated by restricted cubic splines.

The RCS model showed a significant non–linear association between number of teeth and stroke

(*P* = 0.01) (Fig 1) but not with MI (*P* = 0.11) (Fig 2). For stroke, there was stable increased risk between no teeth (edentulousness) and 22 teeth followed by a sharp significant decrease risk up to 32 teeth (Fig 1).

Our sensitivity analyses showed that the estimates for both MI and stroke were only slightly attenuated when full edentates were excluded. Our result also showed that diet did not mediate the observed associations (data not shown).

### Type 2 diabetes mellitus and Cancer

There was also no significant interaction between number of teeth number of teeth and age group, sex, BMI group, smoking status, history of periodontitis and bone loss in the mouth for T2DM and cancer, as well as prevalentT2DM for cancer. We also observed no significant differences for the risk of T2DM and cancer in other number of teeth groups when compared to participants with 28–32 teeth (Table 3) and no linear trend in all models.

**Table 3:** Multivariate hazard ratio and 95% confidence interval of association between number of teeth, type 2 diabetes mellitus and cancer.

Number of teeth				Type 2 diabetes mellitus					
HR per number of tooth			Groups	Incident cases	Model 1	Model 2	Model 3	Model 4	Model 5
	1.00(0.99–1.01)^§^		28–32	248	HR: Ref. (1.00)	HR: Ref. (1.00)	HR: Ref. (1.00)	HR: Ref. (1.00)	HR^†^: Ref. (1.00)
P–value^*^	0.96		24–27	212	1.05 (0.86–1.28)	1.04(0.85–1.27)	1.04 (0.86–1.26)	1.04 (0.85–1.26)	1.05 (0.86–1.29)
			18–23	174	1.18(0.95–1.46)	1.13 (0.91–1.41)	1.11 (0.9–1.36)	1.1 (0.89–1.35)	1.13 (0.91–1.42)
			1–17	250	0.95 (0.68–1.34)	0.88 (0.63–1.24)	0.86 (0.62–1.21)	0.85 (0.61–1.2)	1.06 (0.85–1.31)
			0	103	1.19 (0.89–1.58)	1.08 (0.81–1.44)	1.04 (0.79–1.39)	1.04 (0.78–1.36)	0.98 (0.73–1.33)
			P–value ^‡^		0.19	0.71	0.89	0.93	0.92
Number of teeth				Cancer					
HR per number of tooth			Groups	Incident cases	Model 1	Model 2	Model 3	Model 4	Model 5^†^
	1.00(0.99–1.01)^§^		28–32	299	HR: Ref. (1.00)	HR: Ref. (1.00)	HR: Ref. (1.00)	HR: Ref. (1.00)	HR^†^: Ref. (1.00)
P–value^*^	0.45		24–27	242	1.06 (0.9–1.26)	1.06 (0.9–1.26)	1.18(0.97–1.44)	1.17(0.96–1.44)	1.02 (0.86–1.22)
			18–23	159	0.9 (0.74–1.09)	0.9 (0.74–1.09)	1.08 (0.84–1.39)	1.13 (0.88–1.45)	0.87 (0.7–1.07)
			1–17	218	0.89 (0.61–1.3)	0.89 (0.62–1.28)	1.25(0.82–1.89)	1.3(0.85–1.98)	0.83 (0.68–1.01)
			0	97	1.03 (0.79–1.34)	1.01 (0.78–1.32)	1.11 (0.81–1.54)	1.14 (0.83–1.56)	1.09 (0.83–1.43)
			P–value ^‡^		0.19	0.34	0.87	0.93	0.92

^§^: Same as in Table 2

Model 1: Same as in Table 2

Model 2: Same as in Table 2

Model 3: Same as in Table 2

Model 4: Model 3+ Three retained factors from factor analysis of 49 food groups

Model 5: Same as in Table 2

HR: Hazard ratio,

* P–value for association;

‡ P–value for linear trend;

† adjusted for competing-risk events

T2DM and cancer: All variables as well as the final model (model 4) showed no evidence that proportional hazard assumption was violated (P = 0.09 and 0.52 respectively).

Table 3 also shows that there was no significant effect of every one extra tooth on risk of T2DM and cancer.

**Fig 3:**
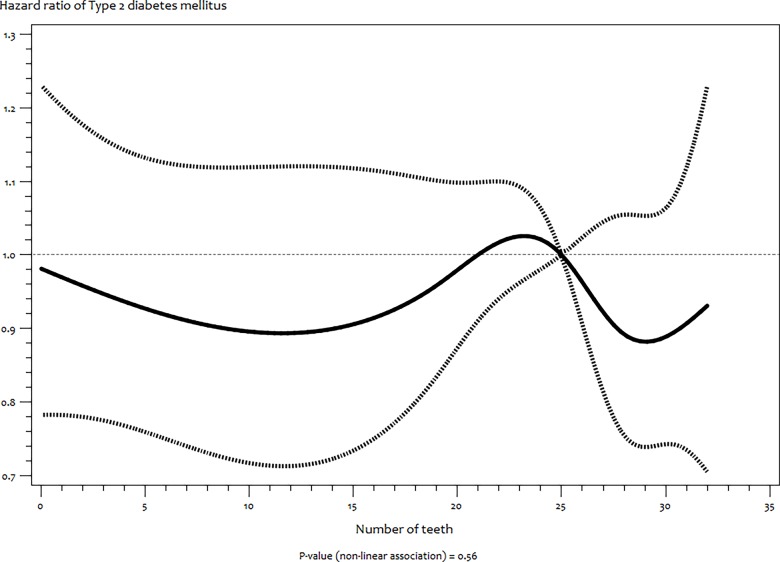
Functional relationship (and 95% pointwise confidence band) between number of teeth and hazard ratios (multivariate adjusted) of incident type 2 diabetes mellitus estimated by restricted cubic splines.

**Fig 4:**
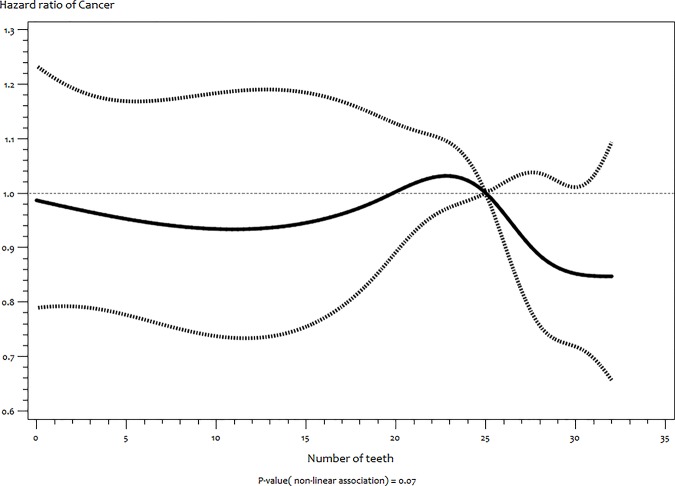
Functional relationship (and 95% pointwise confidence band) between number of teeth and hazard ratios (multivariate adjusted) of incident cancer estimated by restricted cubic splines.

In the same vein, RCS of T2DM (Fig 3) and cancer (Fig 4) also showed no indication of non–linear associations (*P* = 0.56 and 0.07 respectively).

When we excluded full edentates from all models of T2DM and cancer, there was very slight attenuation of HR which was still non–significant (data not shown). Diet did not mediate the observed associations as well (data not shown).

## Discussion

In this retrospective cohort study among middle–aged Germans, lower number of teeth is associated with higher risk of MI independent of smoking, SES, diet and other risk factors of MI. Furthermore, increasing numbers of teeth showed linear association with reduced risk of MI, a non–linear relationship with stroke and no association with T2DM and cancer. Our findings regarding MI is consistent with other cohort studies [8, 14, 26], a meta–analysis of prospective studies [27] and a recent consensus report [28]. The wider confidence interval in our study compared to Hung et al [26] could be due to our relatively small sample size and fewer cases of MI. Our results contrast more recent studies among other European populations [17, 18] that were not included in the meta–analysis by Humphrey et al [27] possibly due to the smaller sample size and shorter follow–up of these studies. The relationship between the number of teeth and MI which is not modified by history of periodontitis and bone loss is consistent with other studies [26]. The relatively large sample of our study would have afforded detection of this effect modification, even if it is small. However, this might suggest a poor validity of self–reported periodontitis is our study population just as it was observed in other German populations [29].

In stroke, the non–significant effects observed in most teeth groups, the greater attenuation compared to the MI models after adjustments and the existence of competing risks indicates less strong association between number of teeth and this disease. However, our results showing non–linear association between numbers of teeth and stroke suggests that this finding should be further investigated as previously proposed [17]. On differentiating the stroke cases in our cohort we found that our observed association is with ischaemic stroke. This is in support of the findings of Joshipura et al [11] but is in contrast to a more recent study [17].

The number of participants with stroke and MI in our study (225 versus 233) is comparable; this could not have affected the possibility to detect significant and similar relation between the number of teeth and these two endpoints. Hazard ratio for all groups with less than 28 teeth were lower for stroke compared to MI (except for those with 1–17 teeth) despite a fairly similar number of cases in each group and adjustment sets. This indicates that our finding across disease endpoints is not biased by the number of cases or type of adjustment. As suggested by Holmlund et al [30] HR above 1.5 (except for those with 1–17 teeth) for MI indicates that our results are not biased by misclassification of individuals or residual confounding. The lower risk in participants with 1–17 teeth compared to those with more than 28 teeth might suggest a significant impact of conditions in early childhood on number of teeth that is also associated with reduced risk of some adverse events in adulthood [31] and number of teeth being an indicator of lifetime oral health is associated with some socioeconomic conditions that is specific to this groupOur findings also revealed that the association between the number of teeth and MI or stroke is not dependent or explained by diet. This suggests that diet has small relevance, if at all, in association between number of teeth and these end points [8].

Our study found no significant association with T2DM. This supports established evidence that uncontrolled T2DM is more likely to result into tooth loss than for reduced number of teeth to precede the onset of T2DM [32]. We also found no association between number of teeth and overall cancer. However, we cannot rule out that the association might be with specific cancer sites [2, 13, 33]. Furthermore, due to tooth loss being highly associated with SES and access to dental care, some authors have proposed that SES might be a stronger predictor of cancer when compared to the number of teeth [34].

A possible biological explanation for the relationship between the number of teeth and MI is that locally synthesized inflammatory cytokines that result in periodontal tissue breakdown before and during tooth loss which reduces number of teeth also propagates systemic dissemination of oral pathogenic bacteria and bacterial products in the blood stream. This leads to increased expression of inflammatory, chemotactic and prothrombotic mediators which promote MI [35]. Early oral dysbiosis that eventually results in tooth loss might also initiate systemic autoantibodies that also promote cardiac dysfunction [36]. Genetic defects in recognition and response to oral pathogenic bacteria [37] and pro–inflammatory genetic factors [8] could also be some explanation for our findings.

The strengths of our study are the large sample size, relatively long follow–up, multiple chronic disease end points and adjustment for several confounders. Our study also investigated the role of diet. Our study has major limitations as the observational design does not allow inferences concerning causality. Self–reported number of teeth information was not validated. Misreporting of the number of teeth due to dental implants, fixed prosthetics and radixes is possible, though this should be very low in our study considering we asked for the number of natural teeth. Professional verification of this reported number of teeth [29] would have validated our assessment. The retrospective extrapolation of the number of teeth may have also biased point estimates due to less exact data. The cross–sectional evaluation of teeth status lacks the exact timing of tooth loss with the subsequent uncertainty of the correct duration of exposure. The application of more valid measure of dental status such as clinical periodontal assessments in large epidemiological studies like ours is limited due to diagnostic procedures that are not straightforward and sometimes cumbersome [29]. Lack of information on actual use of dental care and life–course socioeconomic position could have an impact on teeth number and early diagnosis of diseases. Other bias in our study is that outcomes could have been underreported among individuals with serious and debilitating events. Finally, the use of phone interviews in order to obtain physician confirmation of chronic diseases could also raise some potential of underreporting.

## Conclusions

Despite the limitations and biases stated above, our results convincingly showed an association between number of teeth and specifically myocardial infarction. This study further strengthens the link between oral health and CVD.
